# Appraisal of groundwater susceptibility to pollution using hybrid framework of stable isotopes and indexed approach

**DOI:** 10.1038/s41598-025-30972-9

**Published:** 2025-12-18

**Authors:** Kamilia Hagagg, Nadia Mahmoud Sirag

**Affiliations:** Nuclear and Radiological Research Centre, Egyptian Atomic Energy Authority (EAEA), Cairo, Egypt

**Keywords:** Groundwater vulnerability, Hazard identification, Isotope hydrology, RS/GIS, Environmental sciences, Hydrology, Natural hazards

## Abstract

This study presents an integrated framework for assessing groundwater contamination risks by combining intrinsic aquifer vulnerability, isotope hydrology, and geospatial pollutant loading indicators. A two-dimensional vulnerability mapping approach was developed using a modified GOD index and δ^1^⁸O stable isotope tracer, enabling evaluation of both vertical and lateral contaminant transport within the aquifer system. Pollution sources and loading potential were identified through remote sensing, land use classification, and hydrochemical data interpretation. Groundwater samples revealed significant hydrochemical variability, influenced by anthropogenic activities, salinization, and geogenic processes. Principal Component Analysis (PCA) extracted five dominant components, geogenic, anthropogenic, salinity-driven, redox-related, and agricultural, explaining spatial and temporal patterns of groundwater quality deterioration. δ^1^⁸O values ranged from 5.89 to  -8.62‰, indicating diverse recharge mechanisms and pollutant retardability, and offering insights into groundwater/surface water interactions and lateral migration risks. GOD index values ranged from 0.04 to 0.28, classifying the area predominantly within negligible to medium vulnerability zones. Clay-rich sectors exhibited high natural protectability, while industrial impacted zones showed increased susceptibility. A fuzzy logic-based correlation between GOD scores and isotopic interpretations enhanced aquifer vulnerability assessment, providing a spatially explicit and nuanced risk evaluation. This study integrates established tools to improve groundwater risk characterization. The proposed framework supports contamination threat anticipation, prioritization of high-risk zones, and site-specific protection strategies. It facilitates evidence-based land use planning, targeted monitoring, and adaptive management, contributing to the long-term sustainability of aquifer systems in environmentally complex and industrially stressed regions.

## Introduction

Groundwater plays a pivotal role in addressing global challenges such as land use change, population growth, environmental degradation, and climate change. As a vital resource, it enhances adaptive capacity and mitigates the impacts of diverse human activities^1^. Assessing groundwater quality is essential for the efficient management and sustainable utilization of this resource. High-quality groundwater supports long-term viability, reducing future burdens such as resource depletion, increased treatment costs, and higher abstraction expenses^2^.

Despite the natural filtration capacity of the unsaturated zone, groundwater remains vulnerable to contamination from both geogenic and anthropogenic sources. Naturally occurring trace metals, such as iron and manganese, can dissolve into groundwater and accumulate to concentrations exceeding potable thresholds. Anthropogenic pressures, including waste disposal, excessive abstraction, urban expansion, agricultural runoff, and industrial effluents, significantly compromise groundwater quality^3^. Contaminants from chemical spills, leaking petroleum infrastructure, and widespread fertilizer and pesticide use can percolate through soil layers, reaching the saturated zone and impairing water quality. Additionally, septic systems and poorly managed waste disposal facilities may introduce microbial pathogens, further deteriorating groundwater integrity.

The convergence of pollutants from multiple sources intensifies contamination severity, posing serious risks to aquatic ecosystems, public health, and drinking water reliability. Although many aquifer systems exhibit natural resilience through buffering and attenuation, this capacity is limited. Once polluted, groundwater remediation is often technically complex, financially burdensome, and time-consuming.

Protecting groundwater resources is critical for maintaining ecological integrity and ensuring sustainable water availability. Escalating anthropogenic pressures and climate-induced impacts necessitate proactive management strategies, not only to mitigate contamination risks but also to promote long-term sustainability. The global emphasis on the United Nations Sustainable Development Goals (SDGs), particularly SDG 6 (Clean Water and Sanitation), has elevated the importance of groundwater conservation and integrated resource governance. Between 2020 and 2025, approximately 4748 scholarly publications indexed in the Springer Nature database have addressed various aspects of groundwater vulnerability. This surge reflects a concerted scientific effort to enhance understanding, support evidence-based policymaking, and formulate integrated protection strategies aligned with global sustainability frameworks.

Aquifer vulnerability mapping is a key tool for assessing groundwater susceptibility to contamination. These maps incorporate environmental parameters, such as hydrological conditions, geological structure, hydrogeological behaviour, and soil properties, to identify high-risk zones. They inform land-use planning, guide environmental monitoring, and support the implementation of protective measures.

Although the terms *groundwater susceptibility* and *vulnerability* are sometimes used interchangeably, they have distinct meanings and applications^1^. *Susceptibility* refers to the intrinsic characteristics of a groundwater system that influence its sensitivity to contamination. It evaluates natural factors such as soil type, aquifer lithology, and hydraulic conductivity, independent of pollutant sources. In contrast, *vulnerability* encompasses both susceptibility and the likelihood of contaminants entering the groundwater system. It integrates human activities, pollutant types and sources, and natural variables, offering a more comprehensive risk assessment^2,4^.

Understanding this distinction is essential for effective contamination prevention and resource management. Susceptibility provides insight into natural aquifer defences, such as impervious layers or water table depth, while vulnerability offers a dynamic perspective that accounts for land use, pollutant distribution, and contamination pathways. Strategic planning relies on both concepts to identify high-risk areas and prioritize interventions.

Groundwater contamination prevention remains a central objective in water resource preservation. Current approaches overlay vulnerability assessments with spatial distributions of pollution sources to identify critical zones^5,6^. This framework is informed by ongoing debates surrounding pollution sources, background values, and anti-pollution efficacy^7,8^. Targeted management practices across industrial, residential, and agricultural domains are essential to minimize contamination risks and safeguard groundwater for future generations.

Groundwater vulnerability assessment encompasses a range of methodologies, broadly categorized into overlay/indexing techniques, process-based simulation models, and statistical approaches^9^. Since 2020, Egypt has seen a notable rise in groundwater vulnerability research, with 386 studies published to date (Springer Nature database). Many focus on coastal zone susceptibility to seawater intrusion, reflecting the region’s unique hydrogeological challenges^10–14^. Urbanization, petroleum activities, industrial development, and agriculture continue to exert pressure on groundwater systems, increasing vulnerability^15^. Shallow aquifers, critical water sources in arid and semi-arid regions, are particularly at risk as population growth and land development intensify. Addressing these risks requires a clear understanding of contamination pathways and source dynamics^4,15–17^.

Groundwater vulnerability evaluation is inherently qualitative and relative^18,19^. Among the many assessment methods, three widely recognized approaches are SINTACS^20–23^. DRASTIC is the most globally applied and has undergone several modifications to enhance its effectiveness^24–26^. The GOD method, developed in England in 1987, is particularly suited for data-scarce regions. It employs an empirical framework based on three parameters: groundwater occurrence (G), aquifer lithology and porosity (O), and depth to water table (D). Each parameter is scored from 0.0 (negligible vulnerability) to 1.0 (extreme vulnerability).

This empirical approach enables practical vulnerability mapping and identification of high-risk zones. Its simplicity and minimal data requirements make it valuable in regions with limited hydrogeological information. Groundwater contamination risk refers to the probability that human activities will pollute groundwater to unacceptable levels. It arises from the interaction between intrinsic vulnerability and pollution loading^27^. Vulnerability reflects aquifer characteristics, while pollution loading quantifies the potential contamination from human activities. Notably, pollution loading is dynamic and varies with vulnerability^28^. In contaminated catchments, the hazard represents the maximum potential risk, independent of subsurface attenuation. This underscores the importance of including both point and non-point sources in pollutant loading assessments. The impact of pollution sources depends on toxicity, environmental attenuation, release quantity, and the presence of protective measures^29^. To address these complexities, this study evaluates pollutant toxicity, release probability, and anticipated release volumes.

The primary objective of this paper is to present an integrative framework for assessing groundwater susceptibility to pollution, particularly in regions affected by petroleum-related activities and other anthropogenic stressors. This approach combines stable isotope data with hydrogeological parameters to evaluate vulnerability both vertically and laterally. It further incorporates pollution loading criteria, such as hazard type, pollutant toxicity, and anticipated mobility zones, to support a comprehensive understanding of contamination risks. While the methodology builds upon established tools including the GOD index, fuzzy logic, and isotopic tracing, its contribution lies in the strategic integration of these components into a unified assessment model. By bridging scientific techniques with practical applications, the framework offers a practical basis for groundwater protection and informed decision-making in vulnerable hydrogeological settings.

### Site characteristics

**Fig. 1:**
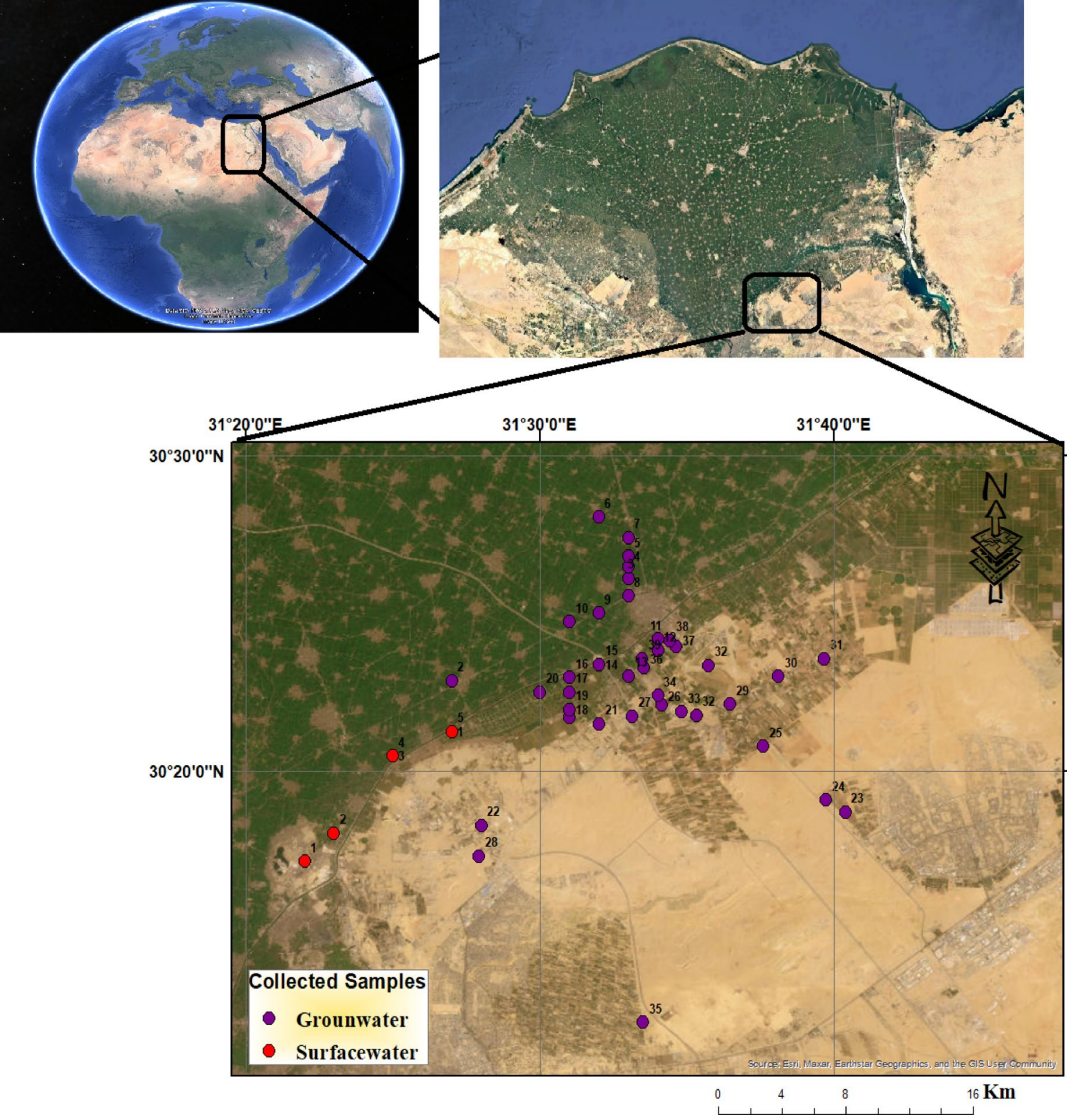
Location map of the study area with collected samples, (the Map was created using (the Map was created using QGIS (QGIS Development Team, 2025, Version: 3.34.6-Prizren. Available at: https://qgis.org).

The north-eastern region of Greater Cairo, Fig. 1, hosts one of Egypt’s major petroleum processing hubs, comprising large-scale refining infrastructure and associated industrial activities. These facilities produce a range of petroleum derivatives including diesel, jet fuel, and liquefied gases, and are strategically positioned to serve the energy demands of the capital and surrounding governorates. However, their proximity to shallow aquifer systems may present significant environmental challenges. Possible potential risks might include leakage from pipelines and storage units, surface runoff from industrial operations, and atmospheric deposition of pollutants, all of which contribute to diffuse contamination pathways,^30–34^.

**Fig. 2:**
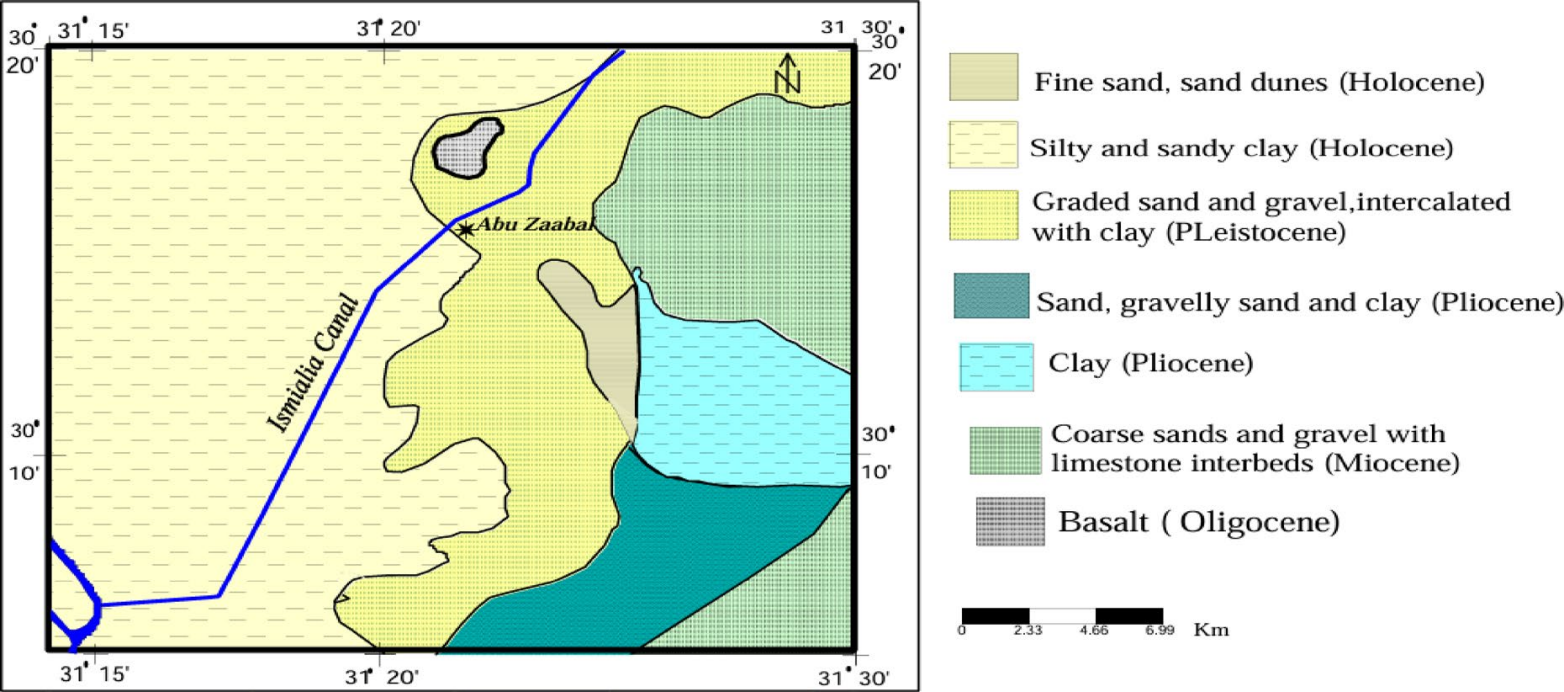
Geological map of the study area^30^.

The study area, covering approximately 2550 km^2^, is situated southeast of the Nile Delta, northeast of Cairo, and south of Belbeis City. It lies between latitudes 30° 10′ and 30° 30′ N and longitudes 31° 20′ and 31° 46′ E. The region is bordered by the Ismailia Canal to the north and the Cairo-Ismailia desert route to the south. The Ismailia Canal divides the area into two parts: the western and north-western segment consists of old agricultural lands, while the eastern and south-eastern section includes desert areas. According to El Sayed^35^, the terrain features mild to moderate slopes, with dry wadis dissecting the surface and sloping toward the north and west. Soil types vary, including sandy clay near cultivated areas, calcareous soils in South El Asher, and sands and gravels between Belbeis and El Asher, Fig. 2.

The Ismailia Canal plays a vital role in Egypt’s water supply and agricultural activities, providing drinking water to villages near the Suez Canal. Located around 125 km north of Cairo and east of the Nile, the canal has an average water flow of 0.28 m/s, depths ranging from 1 to 3 m, and widths between 30 and 70 m^30,36^. It hosts diverse geological formations, including sand and marl on the downstream east bank and silt and mud on the upstream west bank^37^. Approximately 12 million Egyptians depend on the canal for agriculture and drinking water.

The study area encompasses several prominent geomorphological features, including the Nile Delta Flood Plain, El-Khanka and Gebel-Asfr Dunes, Heliopolis Basin, and Gebel El-Hamza Ridge, characterized by fault-related block morphology. Basalt rocks are exposed along fault plains, while Quaternary sediments from the Pleistocene and Holocene dominate the surface, Fig. 2. Miocene and Pliocene sediments outcrop at the eastern sections of Abu Zaabal Quarries, alongside Upper Oligocene basaltic rocks^35^.

**Fig. 3:**
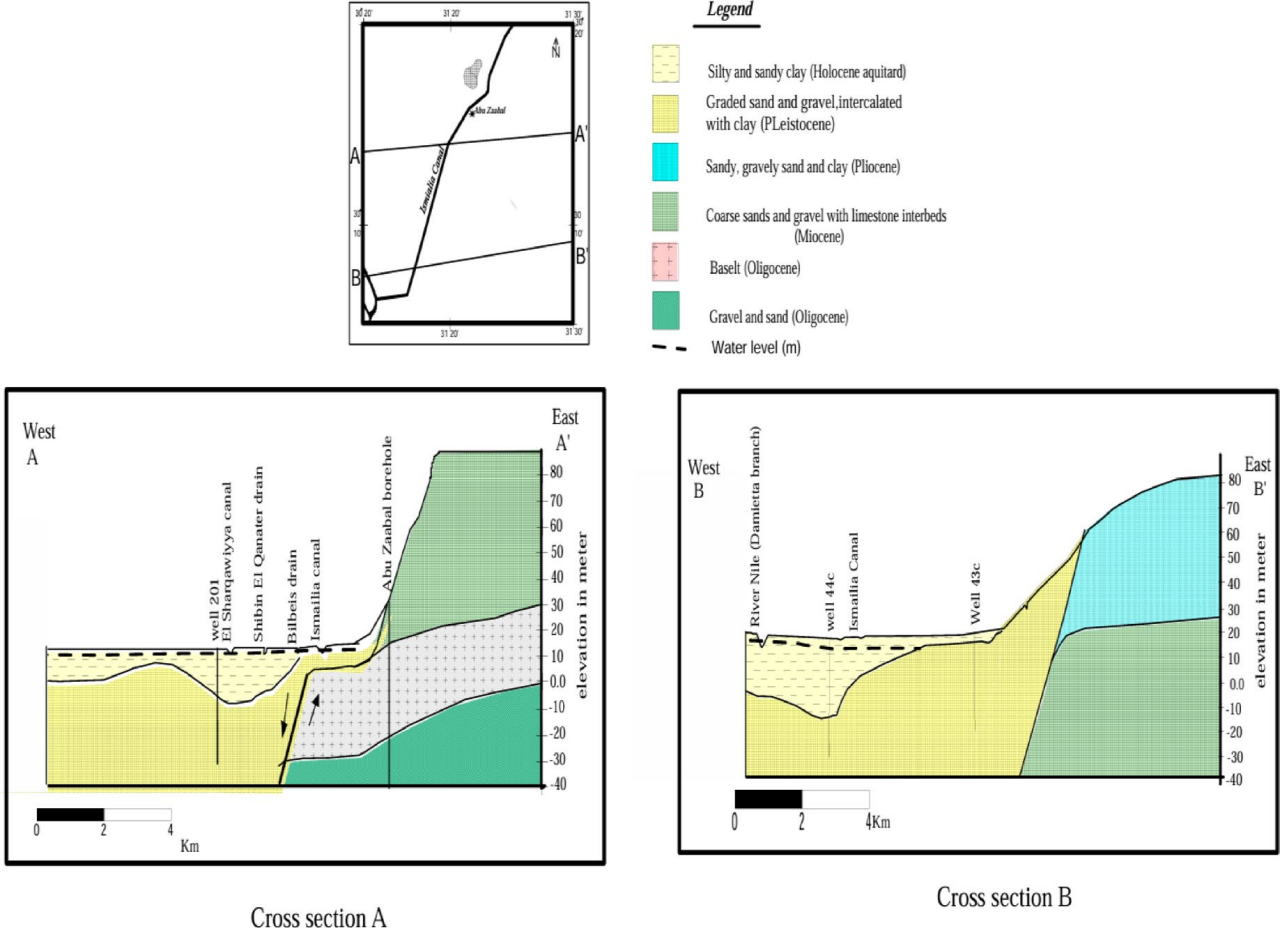
Hydrogeological cross section at various location,^30^.

Groundwater resources in the region are hosted within Quaternary and Miocene aquifers, Fig. 3. The Quaternary aquifer exhibits semi-confined conditions near Belbies and unconfined conditions near Inshas due to the presence of a clay layer. This aquifer is replenished primarily by the Nile water system, including the Ismailia Canal and irrigation return water, with a hydraulic gradient of 50–60 cm/km. The Miocene aquifer varies between unconfined and semi-confined conditions, depending on clay intercalation, with depths ranging from 160 to 240 m along the Cairo-Ismailia desert route^38,39^.

## Experimental and methodologies

### Sampling strategy and analytical procedures

A systematic sampling approach was employed to capture the spatial variability in water quality, as well as, hydrogeological variability, land use diversity, and pollution pressure gradients across the study area. Thirty-nine groundwater wells and five surface water sites were selected to represent a range of aquifer conditions, including variations in lithology, water table depth, and proximity to contamination sources such as industrial zones, agricultural fields, and urban settlements. Surface water samples were collected from recharge interfaces and irrigation return flows to assess interactions with the groundwater system.

Sampling locations were distributed across upstream, midstream, and downstream sectors of the Quaternary aquifer, incorporating both clay-rich protective zones and permeable sectors with probably elevated vulnerability. Site selection was informed by remote sensing land use classification, historical water quality records, and field reconnaissance, ensuring inclusion of both high-risk and naturally protected zones.

Samples were collected in February 2024. Permission was obtained from landowners prior to sampling and no protected areas were accessed. Geographic coordinates (latitude, longitude, elevation) were recorded using a GARMIN eTrex GPS device. Approximately 500 mL of groundwater was collected in clean plastic bottles for hydrochemical analysis. In situ measurements included TDS, pH, EC, and HCO_3_^−^. For trace elements (B, Cd, Cu, Mn, Ni, Pb, Zn, Fe), samples were acidified with nitric acid, filtered, and stored in dark glass bottles. Analyses followed APHA protocols^40^.

Stable isotope samples for δ1⁸O and δ2H were collected in 50 mL airtight plastic bottles, minimizing fractionation. Laser spectroscopy (Piccaro Model 2120i) was used for isotope ratio determination, with results expressed in δ notation (‰) relative to VSMOW. Calibration ensured precision within ± 0.1‰ for oxygen and ± 0.6‰ for hydrogen.

### The groundwater system’s vulnerability

The GOD vulnerability method is an established approach for evaluating groundwater susceptibility to contamination (Foster, 1998). It systematically assesses three primary parameters: Groundwater confinement (G), Overlying strata characteristics (O), and Depth to the water table (D). Each factor is assigned a numerical weight based on its influence on groundwater protection. The GOD method offers a rapid, cost-efficient, and practical assessment for groundwater management and pollution control. The key parameters of this mapping process involves calculating the GOD index through three steps: (i) determining aquifer confinement for the groundwater occurrence parameter (G); (ii) assessing lithological characteristics of the overlying layer; and (iii) assigning ratings for the depth to the water table. The GOD index is derived by multiplying these factors, yielding values between 0 and 1. Importantly, the final index typically reflects the lowest score assigned to any of the three parameters, ensuring that the most protective factor determines the vulnerability rating. For example, if two parameters receive the highest rating (1.0), the index is determined by the third parameter’s score index were retrieved from El Sayed’s^35^, the data was interpolating in QGIS (QGIS Development Team, 2025, Version: 3.34.6-Prizren. Available at: https://qgis.org).

The final integrated vulnerability index was composed from three thematic layers for 42 boreholes as following.

1. *Groundwater Confinement (G)* that classifies the aquifer as confined, semi-confined, or unconfined, indicating its exposure to external contaminants.
2. *Overlying Strata (O)* that examines the lithological composition and permeability of surface materials, which serve as protective layers.
3. *Depth to Water Table (D)* that quantifies the vertical distance between the surface and groundwater, affecting the potential for pollutant infiltration.

These factors are assigned numerical values, integrated into a GOD vulnerability index, and categorized into three levels: low, moderate, and high vulnerability according to (Foster, 1998). Confined aquifers with protective overlying layers and deep-water tables exhibit low vulnerability, while unconfined aquifers with permeable strata and shallow water tables are classified as highly vulnerable to contamination. This classification system supports groundwater protection strategies by aiding in risk assessment and sustainable management practices.

### GIS interpolation for thematic mapping and fuzzy logic approach

Geographic Information Systems (GIS) facilitate interpolating areal distribution for thematic mapping, enabling spatial analysis and visualization of environmental and geospatial data. Interpolation techniques, such as Inverse Distance Weighting (IDW), Kriging, and Spline, estimate values at unsampled locations, enhancing the accuracy of thematic maps in hydrology, climate studies, and land-use planning. In this work Inverse Distance Weighting (IDW), It estimates unknown values based on the weighted average of nearby known points, assuming that closer points have a greater influence than distant ones. It is particularly effective in cases of irregularly distributed data, ensuring a smooth and coherent representation of groundwater characteristics. Several advantages of IDW include its computational efficiency, its ability to preserve local variability by emphasizing nearby data points, and its flexible weighting parameters, allowing for spatial customization. These attributes collectively enhance environmental monitoring and support groundwater resource management, facilitating precise spatial analysis for sustainable water systems.

Additionally; the Fuzzy logic approach improves GIS interpolation by incorporating uncertainty and imprecision,^41,42^. By assigning membership values rather than fixed classifications, fuzzy interpolation enhances spatial accuracy, supports multi-criteria decision-making, and improves thematic map reliability. Integrating fuzzy logic into GIS-based interpolation methods allows for more flexible and realistic spatial representations, optimizing environmental analysis and resource management. The fuzzy logic process consists of four sequential stages. Fuzzification transforms precise input values into fuzzy values through predefined membership functions. Rule evaluation applies a set of fuzzy rules to the fuzzified inputs, deriving corresponding fuzzy outputs. Aggregation integrates these fuzzy outputs into a unified fuzzy set. Finally, defuzzification converts the aggregated fuzzy set into a crisp numerical output, facilitating decision-making and real-world application,^42^.

#### Fuzzy logic-based vulnerability assessment

To enhance the spatial resolution and interpretive capacity of aquifer vulnerability analysis, a fuzzy logic model was developed to integrate intrinsic vulnerability scores derived from the GOD index with isotopic indicators of recharge and pollutant migration, specifically δ^1^⁸O values. This approach enables the representation of uncertainty and gradual transitions in vulnerability classification, overcoming the limitations of discrete threshold-based models.

##### Input variables and membership functions

Two input parameters were selected for the fuzzy inference system: (1) GOD index values representing intrinsic aquifer vulnerability, and (2) δ^1^⁸O stable isotope values reflecting recharge source characteristics and potential anthropogenic influence.

Trapezoidal membership functions were defined for each input variable:

- **GOD Index**:
  o Negligible vulnerability: 0.00–0.10
  o Low vulnerability: 0.10–0.20
  o Medium vulnerability: 0.20–0.30
- **δ**^**1**^**⁸O Values**:
  o Depleted signature: -9 to -4‰ (meteoric recharge)
  o Transitional signature: -4 to + 2‰ (mixed recharge)
  o Enriched signature: + 2 to + 6‰ (surface water and anthropogenic influence)

##### Rule base and inference structure

A set of fuzzy rules was constructed to define the relationship between input variables and the output vulnerability classification. Examples include:

- IF GOD is Medium AND δ^1^⁸O is Enriched THEN vulnerability is High
- IF GOD is Low AND δ^1^⁸O is Transitional THEN vulnerability is Moderate
- IF GOD is Negligible AND δ^1^⁸O is Depleted THEN vulnerability is Low

These rules were designed to reflect both vertical protectability and lateral migration potential within the aquifer system.

### Principal component analysis (PCA)

Principal Component Analysis (PCA) was employed to identify the dominant factors controlling groundwater and surface water quality and to reduce the dimensionality of the multivariate dataset. The analysis included 20 variables measured across all collected samples: aluminium (Al), cadmium (Cd), cobalt (Co), vanadium (V), nickel (Ni), molybdenum (Mo), chromium (Cr), iron (Fe), chloride (Cl^−^), magnesium (Mg^2+^), sodium (Na^+^), total dissolved solids (TDS), electrical conductivity (EC), calcium (Ca^2+^), sulfate (SO_4_^2−^), barium (Ba^2+^), manganese (Mn), bicarbonate (HCO_3_^−^), potassium (K^+^), and zinc (Zn).

Prior to PCA, all variables were standardized using z-score transformation to eliminate scale effects and ensure comparability. The suitability of the dataset for PCA was confirmed using the Kaiser–Meyer–Olkin (KMO) measure of sampling adequacy and Bartlett’s test of sphericity. The KMO value exceeded 0.7, and Bartlett’s test was statistically significant (*p* < 0.001), indicating that the data were appropriate for factor extraction. PCA was performed using the correlation matrix to account for differences in units and variance among the variables. Varimax rotation with Kaiser normalization was applied to maximize the interpretability of the component loadings and minimize cross-loadings. Components with eigenvalues greater than 1.0 were retained according to Kaiser’s criterion. Component loadings greater than ± 0.50 were considered significant for interpretation. All statistical analyses were conducted using SPSS software. The extracted components were used to interpret spatial patterns of groundwater degradation and to support the fuzzy logic–based vulnerability classification.

## Results and discussion

### Geochemical evolution of groundwater system

**Fig. 4:**
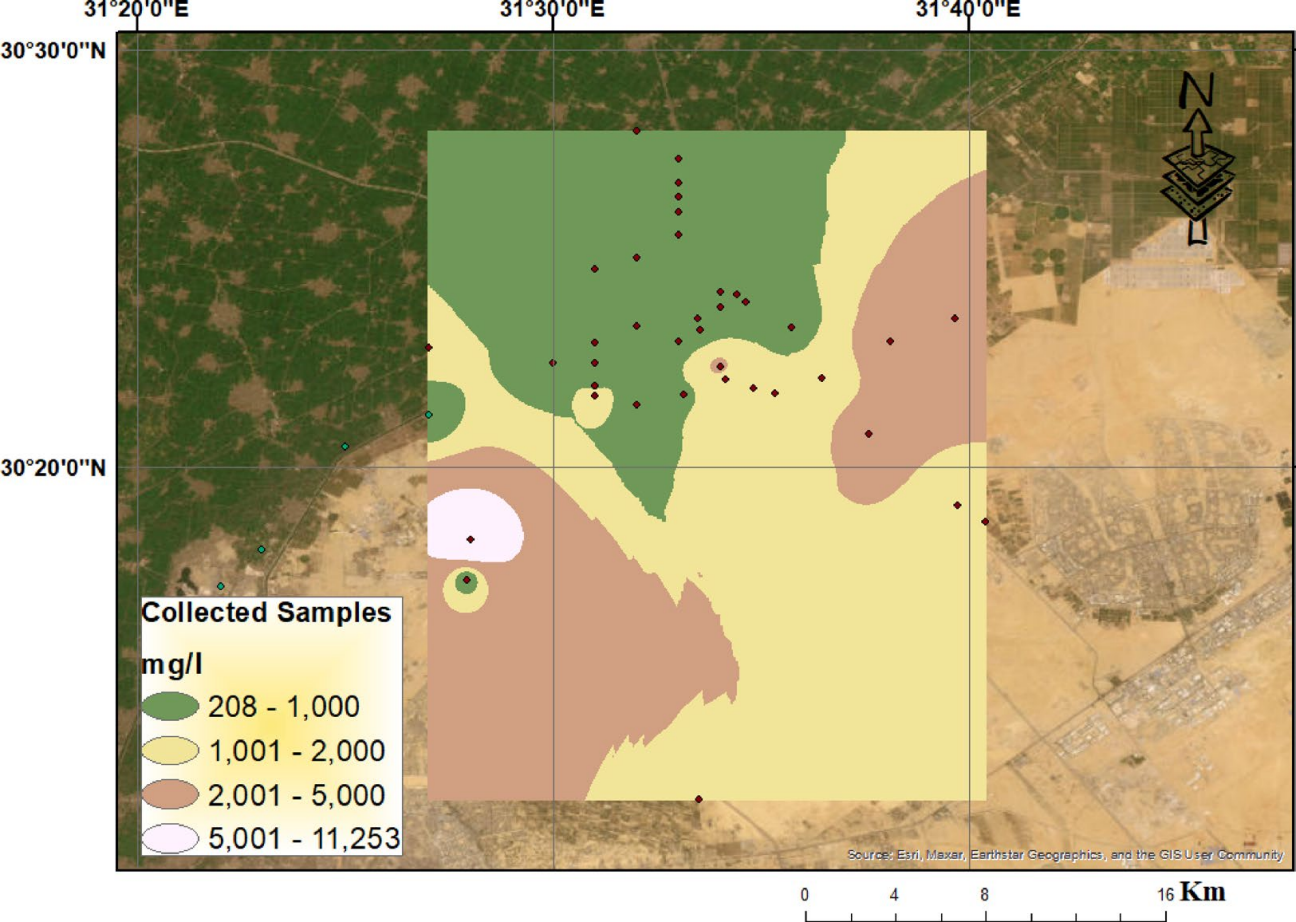
Spatial distribution of TDS (mg/l) in the whole study area (the map was created (the Map was created using QGIS (QGIS Development Team, 2025, Version: 3.34.6-Prizren. Available at: https://qgis.org).

The wide ranges observed in the chemical and isotopic composition of the collected groundwater samples reflect the complex geochemical evolution and heterogeneity of the aquifer system. The pH values, ranging from 7.00 to 8.50, indicate near-neutral to slightly alkaline conditions, consistent with the dissolution of carbonate minerals, as supported by elevated bicarbonate concentrations. Total dissolved solids (TDS) and electrical conductivity (EC) exhibit substantial variability, with TDS ranging from 179 to 11,259 mg/L and EC from 247 to 18,990 µS/cm. These variations suggest differences in mineralization levels, where low values near recharge zones, such as areas adjacent to the Ismailia Canal, reflect fresh water input, while higher values in distal locations are indicative of prolonged residence time, intensified water–rock interaction, and anthropogenic salinization due to irrigation return flows and excessive groundwater abstraction, Fig. 4.

Sodium and chloride concentrations, ranging from 20 to 3000 mg/L and 16.71 to 6170.21 mg/L respectively, show pronounced variability, pointing to both geogenic sources such as halite dissolution and anthropogenic inputs from agricultural and domestic activities. Calcium and magnesium also display wide ranges (7.44–499.70 mg/L and 3.27–434.10 mg/L), suggesting differential dissolution of carbonate and silicate minerals, as well as possible mixing between fresh and saline waters. The stable isotopic composition, with δ^1^⁸O values ranging from − 8.62‰ to + 5.89‰ and δD from − 0.83‰ to + 15‰, reveals significant fractionation. Depleted isotopic signatures are indicative of meteoric recharge, while enriched values reflect evaporative concentration, supporting the presence of multiple recharge mechanisms and varying residence times.

Trace element concentrations further elucidate redox conditions and potential contamination pathways. Elevated levels of iron and manganese, with values reaching up to 0.10 mg/L and 0.18 mg/L respectively, suggest reductive dissolution under sub-oxic conditions, particularly in zones with organic matter or limited flow. The sporadic presence of elements such as zinc, chromium, and lead, although generally low, may reflect localized anthropogenic inputs from agricultural runoff or industrial sources. Collectively, these geochemical indicators demonstrate that the groundwater system is shaped by a combination of natural processes, including mineral dissolution, ion exchange, and aquifer mixing, and anthropogenic stressors such as irrigation and over-extraction. The observed ranges are essential for understanding the spatial variability and geochemical evolution of the aquifer, with direct implications for groundwater quality assessment, vulnerability mapping, and sustainable resource management (Table 1).

**Table 1:** Statistical analyses of major ions and stable isotopic content for the collected samples.

Item	pH	Ec	TDS (mg/l)	Ca (mg/l)	Mg (mg/l)	Na (mg/l)	K (mg/l)	CO_3_ (mg/l)	HCO_3_ (mg/l)	SO_4_ (mg/l)	Cl (mg/l)	δ^2^H (‰)	^18^O (‰)
Mean	7.70	2345.48	1299.80	89.23	38.39	309.76	9.35	13.70	185.29	354.57	393.22	15	− 0.83
STD	0.35	3632.14	1834.43	96.18	65.77	494.87	4.93	16.08	71.86	388.44	971.29	8.52	3.95
Kurtosis	0.33	14.19	23.00	13.76	35.96	23.07	7.25	28.59	2.21	6.72	34.20	− 1.2	− 0.53
Skewness	0.58	3.67	4.42	3.65	5.86	4.41	2.20	4.94	1.22	2.33	5.67	− 3.4E-17	− 0.68
Min	7.00	247.00	179.00	7.44	3.27	20.00	3.00	0.00	82.35	18.93	16.71	1	− 8.62
Max	8.50	18990.00	11259.00	499.70	434.10	3000.00	30.00	105.50	430.05	1964.00	6170.21	29	5.89

**Fig. 5:**
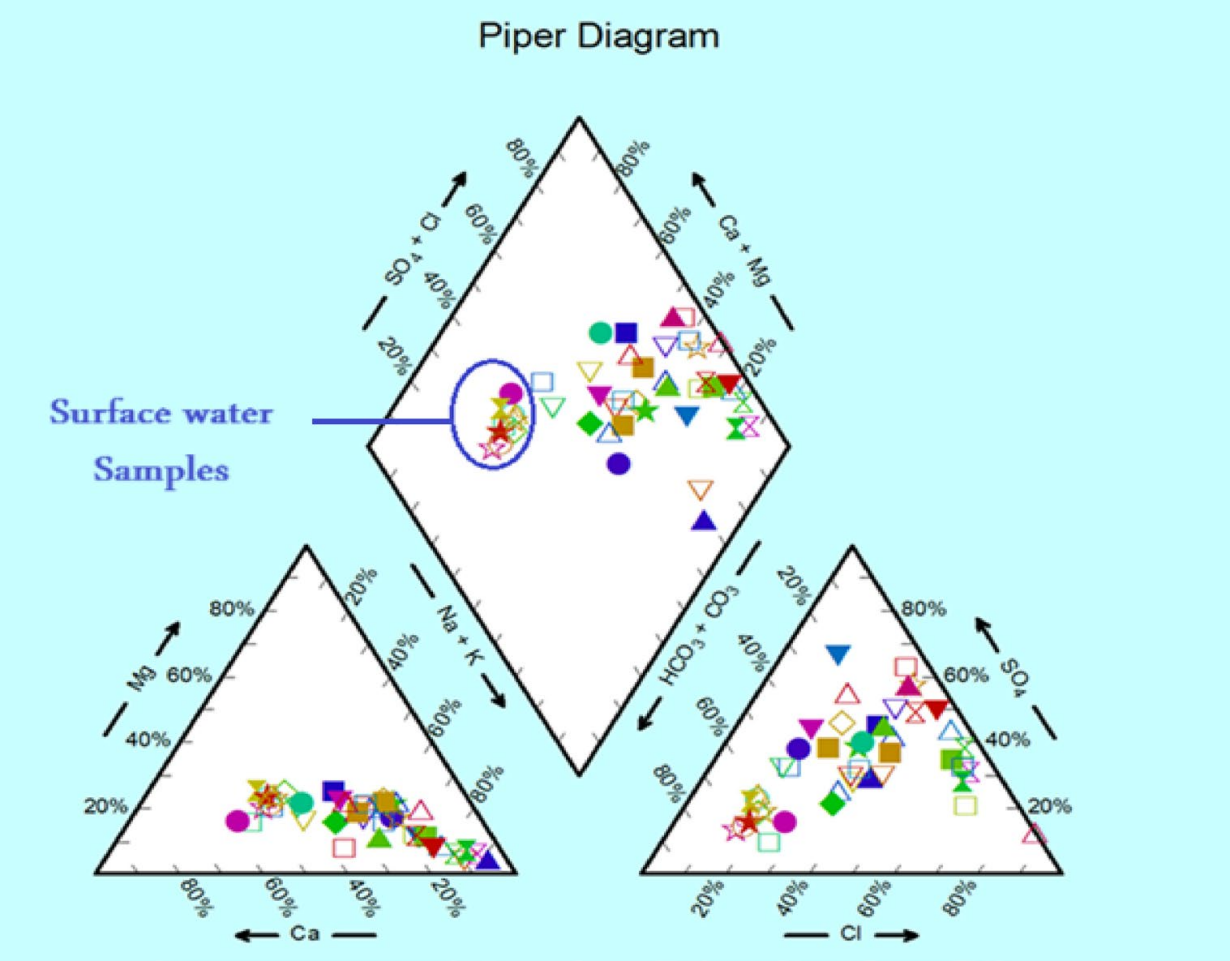
Piper diagram for the collected samples.

Based on the Piper diagram in Fig. 5, the hydrochemical facies of the collected samples are clearly delineated across the cation and anion triangles and projected into the central diamond field. The position of the surface water sample suggests dominance of sodium and chloride ions, consistent with a Ca, Mg–HCO_3_ water type, which may result from active recharge fresh system. On the other hand, groundwater samples, distributed more broadly across the diagram, exhibit varied facies including Ca–HCO_3_, Na–SO_4_, and mixed types. About 60% with a hydrochemical composition of (Na^+^ + K^+^)/(Cl^−^ + SO_4_^2−^) and (Ca^2+^  + Mg^2+^)/(Cl^−^ + SO_4_^2−^). This can be related to cationic exchange on the surface of the clay beds in the aquifer composition. Two other hydrochemical facies ((HCO_3_^−^/(Ca^2+^ + Mg^2+^) and (HCO_3_^−^/(Na^+^ + K^+^)) appear in about 22% of the samples. These are typical meteoric waters that have high Na^+^ concentration due to leaching or cationic exchange. The rest of the samples fall on (SO_4_^2−^ + Cl^−^/(Ca^2+^ + Mg^2+^)) field reflecting the effect of fertilizers application. This diversity reflects the influence of water–rock interaction, recharge variability, and possible contamination or salinization processes. The presence of samples trending toward the Ca–HCO_3_ facies suggests zones of active recharge and carbonate dissolution, while those shifting toward Na–Cl or Na–SO₄ facies may indicate longer residence times, evaporative concentration, or anthropogenic impact.

**Fig. 6:**
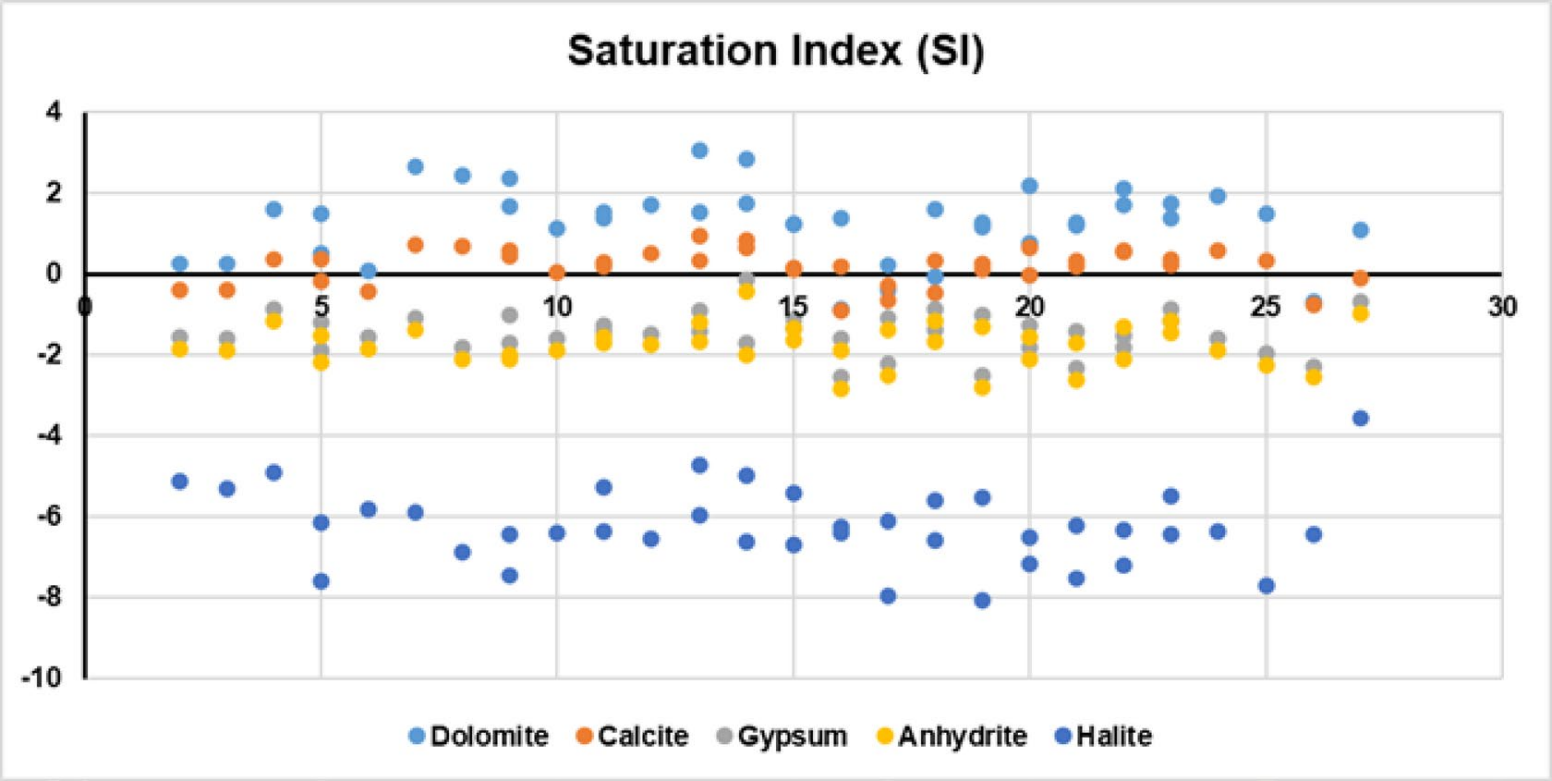
Saturation index (SI) calculted using PHREEQC for the collected samples.

Further geochemical modelling using PHREEQC was applied to exprese the extent to which a groundwater has reached chemical equilibrium with the minerals within the aquifer matrix through the calculation of Saturation index (SI). The saturation index, as indicated in Fig. 6, trends provide valuable insight into the mineral stability and geochemical evolution of the groundwater system. Dolomite and Calcite show multiple data points above the equilibrium line, indicating that groundwater in several locations is supersaturated with respect to these carbonate minerals. This suggests active precipitation processes, likely driven by elevated pH and bicarbonate concentrations, consistent with carbonate buffering and geochemical evolution toward Ca–HCO_3_ facies. Gypsum and Anhydrite exhibit data points near or below the saturation line, implying that these sulphate minerals are generally undersaturated and may be dissolving into the groundwater. This dissolution contributes to elevated SO_4_2^−^ concentrations observed in the chemical dataset and reflects water–rock interaction in sulphate-bearing formations. Halite shows consistent undersaturation across the dataset, indicating that sodium and chloride concentrations are not high enough to reach halite saturation. This supports the interpretation that Na^+^ and Cl^−^ are primarily derived from other sources, such as anthropogenic inputs or cation exchange, rather than direct halite dissolution.

### Groundwater pollution loading in the study area and their risks

Groundwater pollution loading refers to the introduction and accumulation of contaminants in subsurface water systems, influenced by various natural and anthropogenic factors, (Huan etal, 2018). It quantifies the potential impact of pollutants on groundwater quality based on their source, mobility, concentration, and persistence. In this paper the pollution loading is primarily governed by:

1. *Sources of pollution* Includes industrial discharges, agricultural runoff, landfill leachates, mining activities, and urban wastewater infiltration.
2. *Transport mechanisms* Pollutants migrate through the subsurface via advection, dispersion, diffusion, and adsorption, impacting aquifer composition.
3. *Pollutant characteristics* The chemical and physical properties of contaminants, including solubility, degradation rate, and reactivity, determine their persistence and toxicity.
4. *Hydrogeological factors* The vulnerability of an aquifer system depends on soil permeability, groundwater flow dynamics, recharge rates, and geological composition.
5. *Risk assessment* Pollution load risk is evaluated based on contamination probability and intensity, incorporating spatial distribution, hydrological connectivity, and removal processes.

Understanding pollution loading enables effective groundwater protection measures, such as contamination prevention strategies, remediation techniques, and sustainable water resource management.

#### Elucidation of the major pollution sources in groundwater system and their prospective health effects

The dataset presented in Table 2 showed concentrations of various Potential Toxic Elements (PTMs), highlighting potential environmental and health implications. Essential trace elements such as copper (Cu), zinc (Zn), and iron (Fe) are crucial for physiological functions but must remain within safe thresholds to prevent toxicity. The statistical analysis reveals variability in these elements, as indicated by Zn’s high kurtosis (4.44) and maximum concentration (0.20), suggesting localized enrichment that may influence immune function and gastrointestinal health. Lead (Pb), cadmium (Cd), chromium (Cr), and aluminium (Al), recognized for their adverse health effects, exhibit low mean concentrations, but their skewness values (Pb = 1.80, Al = 0.21) highlight distribution asymmetry, indicating occasional peaks in contamination. Iron (Fe) and manganese (Mn), while naturally occurring, demonstrate significant kurtosis (Fe = 11.56, Mn = 37.85), suggesting sporadic extreme values that could pose neurological risks if excessively present in water systems. These statistical findings underscore the necessity of continuous monitoring, as heavy metals such as lead and cadmium, even in trace amounts, can contribute to long-term health issues, particularly affecting cognitive development in children. Assessing these trends in relation to established regulatory limits would provide clearer insights into exposure risks and necessary intervention measures.

**Table 2:** Statistical analyses of trace elements for the collected samples (mg/l).

Item	Al	B	Cd	Co	Cr	Cu	Fe	Mn	Mo	Ni	Pb	V	Zn
Mean	0.02	0.22	0.00	0.00	0.01	0.01	0.02	0.01	0.00	0.00	0.01	0.01	0.03
STD	0.02	0.25	0.00	0.00	0.00	0.00	0.02	0.03	0.00	0.00	0.00	0.00	0.04
Kurtosis	− 2.06	3.79	− 2.06	− 2.06	4.43	− 2.11	11.56	37.85	− 1.66	− 0.88	− 2.06	2.83	4.44
Skewness	0.21	1.97	− 0.21	− 0.21	− 0.76	− 1.04	2.52	6.08	-0.55	0.19	0.21	1.80	1.83
Min	0.00	0.03	0.00	0.00	0.00	0.01	0.01	0.00	0.00	0.00	0.01	0.01	0.00
Max	0.04	1.11	0.00	0.00	0.02	0.01	0.10	0.18	0.01	0.01	0.01	0.03	0.20

Effective environmental management and remediation require the precise identification of contamination sources to mitigate adverse impacts on ecosystems and public health. Principal Component Analysis (PCA) is a multivariate statistical technique commonly employed to reduce the dimensionality of large datasets while preserving most of the inherent variability. By analysing multiple water quality parameters simultaneously, PCA facilitates the identification of contamination patterns and key contributing sources. In this study, PCA was applied to assess the correlation among significant variables in the collected groundwater samples using SPSS 22 software, (Table 3). This method decomposes correlated variables into principal components, ranked by their contribution to total dataset variance. The extracted components provide insights into dominant geochemical processes influencing water quality, aiding in the interpretation of pollution sources and hydrological interactions.

**Table 3:** Principal component analysis of the collected samples.

Item	PC1	PC2	PC3	PC4	PC5
Al	− 0.98				
Cd	0.98				
Co	0.98				
V	− 0.98				
Ni	0.95				
Mo	0.92				
Cr	0.65				
Fe	0.53		0.77		
Cl		0.99			
Mg		0.97			
Na		0.96			
TDS		0.96			
Ec		0.80			
Ca		0.76	0.57		
SO_4_		0.50	0.77		
Ba			0.74		
Mn				0.77	
HCO_3_				0.61	
K					0.74
Zn					− 0.69

According to the Principal Component Analysis (PCA) results from Table 3, the analysis categorizes groundwater quality variables into five principal components (PCs), each reflecting distinct geochemical processes and contamination sources as following.

1. PC1 (Heavy Metal Influence)

This component exhibits strong negative and positive loadings for Al (-0.98), Cd (0.98), Co (0.98), V (− 0.98), Ni (0.95), Mo (0.92), and Cr (0.65). The presence of these elements suggests that PC1 primarily represents geogenic influences, such as natural mineral dissolution, and potential anthropogenic contamination from industrial or agricultural activities. The high positive correlation of Cd, Co, Ni, and Mo indicates possible sources related to fertilizer use, wastewater discharge, or heavy metal leaching from geological formations.

2. PC2 (Salinity and Major Ion Chemistry)

Strong positive loadings for Cl (0.99), Mg (0.97), Na (0.96), TDS (0.96), EC (0.80), Ca (0.76), and SO₄ (0.50) suggest that PC2 is highly associated with salinity variations in groundwater. The dominance of chloride, sodium, and magnesium suggests groundwater interaction with evaporative deposits or saline intrusion from nearby sources. The link between TDS, EC, and major cations (Ca, Mg, Na) confirms the influence of water–rock interactions and anthropogenic activities, such as intensive irrigation and industrial discharge, on groundwater chemistry.

3. PC3 (Localized Geochemical Variability)

Ba (0.74) is the dominant variable in this component, indicating localized geochemical processes that may result from carbonate dissolution or mineral precipitation. This component may reflect natural groundwater evolution, including interaction with carbonate-rich formations, affecting Ba concentrations.

4. PC4 (Redox-Driven Processes)

Mn (0.77) and HCO_3_ (0.61) are the primary contributors to this component, suggesting redox-controlled processes affecting groundwater chemistry. Elevated Mn levels often indicate reducing conditions, which can lead to mobilization of trace elements from sediments into groundwater. The association with HCO_3_ suggests contributions from carbonate weathering or biogeochemical activities, such as microbial reduction reactions.

5. PC5 (Agricultural and Anthropogenic Influence)

K (0.74) and Zn (− 0.69) dominate this component, implying links to agricultural inputs, including fertilizers and pesticides, as well as industrial pollution sources. The presence of potassium suggests agricultural enrichment through fertilization practices, while zinc variability may indicate anthropogenic contamination from urban runoff or wastewater discharge.

The PCA results highlight the complex hydrogeochemical interactions governing groundwater quality in the study area. The influence of geogenic, anthropogenic, salinity-driven, redox-related, and agricultural processes underscores the need for continuous groundwater monitoring to mitigate contamination risks. The findings can be used to develop effective water management strategies, including pollution control measures, aquifer protection policies, and sustainable irrigation practices.

#### Integration of land use and land cover (LULC) and potentially hazardous facilities on groundwater pollution sources

Land use and land cover (LULC) significantly influence groundwater pollution sources by altering hydrological processes, recharge rates, and contaminant transport mechanisms,^43–46^. Urbanization and industrialization contribute to groundwater contamination through increased impervious surfaces, leading to reduced infiltration and higher pollutant accumulation. Industrial discharge and wastewater infiltration introduce heavy metals, nitrates, and organic pollutants into aquifers,^47^. Agricultural activities exacerbate groundwater contamination through excessive fertilizer and pesticide application, resulting in elevated nitrate and phosphate concentrations,^48^. Irrigation practices can increase groundwater salinity, while livestock farming contributes microbial and organic pollutants. Deforestation and land cover changes reduce natural filtration capacity, accelerating sedimentation and pollutant transport. Additionally, mining and landfill operations release heavy metals, leachates, and organic contaminants, leading to long-term groundwater quality deterioration.

**Fig. 7:**
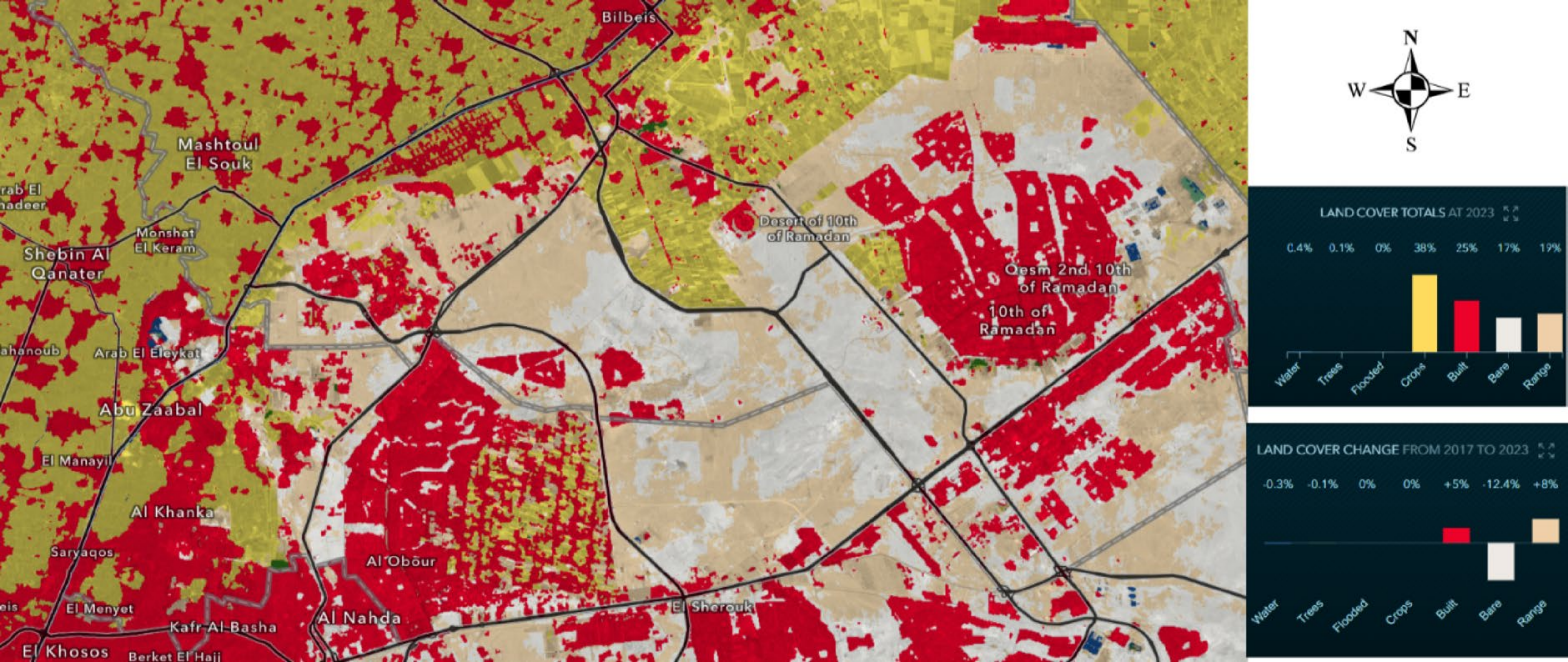
Landuse/land cover 2023 for the study area,^51^.

Understanding LULC impacts on groundwater contamination is critical for sustainable water management, pollution mitigation, and environmental conservation. Integrating hydrological assessments with land use analysis enhances groundwater protection and informs effective contamination prevention strategies. The spectral reflectance characteristics of the study area were utilized to classify land cover and land use types, providing insight into environmental changes affecting groundwater systems. As illustrated in Fig. 7, rapid expansion of the built-up environment is occurring at the expense of agricultural land, vegetation, and water bodies. This shift in land use may impose significant stress on the groundwater system by altering recharge rates and contamination pathways.

The integration of the spatial distribution of potentially hazardous facilities and their respective zones of influence with the land use and land cover classification enhances the assessment of pollution load and groundwater contamination risks. This approach provides a more comprehensive evaluation by linking environmental characteristics with anthropogenic activities that directly affect groundwater quality. According to Zhang^49^, pollution sources are categorized into five primary types: industrial activities, hazardous waste disposal sites, mining operations, landfill areas, and agricultural contamination. Each of these categories represents distinct contamination mechanisms with varying pollutant compositions and degrees of environmental impact,^50^. Industrial activities, would discharge heavy metals, organic solvents, and toxic effluents, while agricultural contamination primarily involves excessive nutrient loading (nitrates and phosphates), pesticide residues, and microbial pollutants. Mining operations might contribute acidic drainage and metal leaching, whereas landfill sites might pose threats through leachate infiltration containing organic pollutants and inorganic contaminants. Given the diverse nature of industrial pollution, the spatial extent of contamination varies depending on site-specific characteristics such as pollutant mobility, soil permeability, and groundwater flow dynamics. To quantify the potential migration and dispersion of contaminants, this study employs buffer zones around pollution sources that was extracted using RS/GIS, as a critical step for assessing pollutant spread and delineating affected regions within the aquifer system. Furthermore, toxicity scores are assigned to pollutants based on their physicochemical properties, degradation rates, mobility, and carcinogenic or non-carcinogenic potential^49^. Toxicity assessments ensure accurate classification of contamination severity and contribute to the formulation of mitigation strategies aimed at groundwater protection, pollutant containment, and long-term environmental sustainability.

**Fig. 8:**
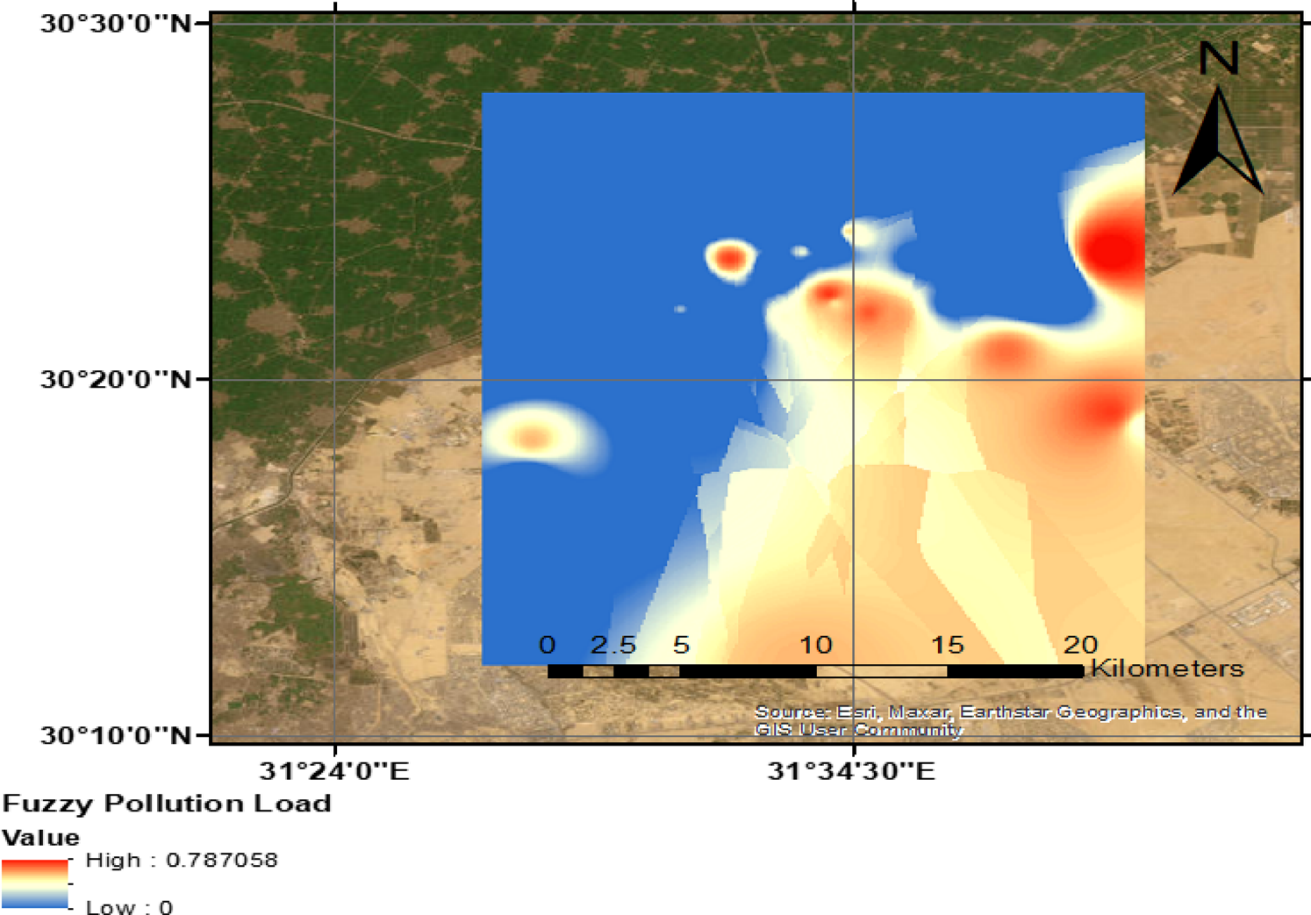
Spatial distribution of pollution load in the study area, (the Map was created using (the Map was created using QGIS (QGIS Development Team, 2025, Version: 3.34.6-Prizren. Available at: https://qgis.org).

Figure 8 represents the spatial distribution of pollution loading across the study area, highlighting the concentration and intensity of contaminants from various sources. The map employs a mosaic grid representation to visualize pollution load contributions from industrial activities, hazardous waste disposal, mining operations, landfills, and agricultural contamination. High pollution load zones are primarily associated with industrial sectors, where the overlap of buffer regions indicates pollutant migration and dispersion. The clustering of pollution sources in densely industrialized areas contributes to elevated contamination risks, particularly in locations with textile, chemical, and petroleum processing industries. Additionally, the convergence of multiple pollution sources exacerbates pollutant accumulation, leading to groundwater vulnerability. The map further demonstrates a spatial disparity in pollution intensity, with the eastern portion of the study area exhibiting a greater probability of pollutant discharge compared to the predominantly agricultural western section. This distinction underscores the role of industrial expansion in groundwater contamination and highlights the necessity for targeted mitigation strategies to safeguard water resources.

This section illustrates the potential causes of groundwater pollution within the research area, focusing on the mechanisms influencing contamination pathways and groundwater system vulnerability. A detailed assessment of pollutant transport routes is crucial to identifying high-risk zones, enabling the formulation of effective mitigation strategies.

### The added value of stable isotopes in susceptibility of groundwater to pollution

Both 1⁸O and 2H are stable isotopes commonly used in hydrogeological studies to assess groundwater recharge, mixing processes, and contamination pathways. Their isotopic ratios provide insights into water origin, movement, and interactions within an aquifer. The variations in isotopic values help distinguish different sources of groundwater recharge, such as precipitation, river infiltration, and irrigation return flow. These isotopes also indicate evaporation effects and climatic influences, particularly in arid regions where isotopic enrichment occurs. Additionally, their distribution aids in identifying mixing between shallow and deep groundwater systems and interactions with surface water inputs. In pollution assessments, 1⁸O and 2H serve as tracers for anthropogenic influences, such as wastewater infiltration and agricultural runoff. By comparing isotopic signatures with known reference sources, researchers can evaluate groundwater vulnerability and develop effective water management strategies.

Groundwater susceptibility and vulnerability indices effectively trace an aquifer’s ability to attenuate pollution from surface sources through the unsaturated zone in a vertical dimension. However, contamination risks are not solely confined to vertical transport; lateral migration can occur due to surface water-groundwater interactions or hydraulic connectivity between adjacent aquifers. To enhance the evaluation of potential lateral migration and linkages, stable isotopes were incorporated as additional indicators. Isotopic signatures provide valuable insights into hydrodynamic exchange and water mixing processes, allowing for a more comprehensive assessment of contamination pathways. By analysing the distribution of isotopes such as oxygen-18 (1⁸O) and deuterium (2H), researchers can trace water movement and identify possible cross-flow between interconnected aquifers. This isotopic approach improves the understanding of groundwater system dynamics and refines vulnerability assessments by capturing both vertical infiltration and horizontal pollutant transport mechanisms.

The observed range of δ1⁸O values in the study, (Table 1), are between 5.89 and − 8.62‰ indicates variations in recharge mechanisms and differing retardability of pollutants within the aquifer system. These isotopic signatures reflect interactions between groundwater and surface water, offering insights into contamination risks associated with lateral pollutant migration. The wide range of δ1⁸O values suggests a heterogeneous recharge environment, influenced by both natural and anthropogenic processes. Elevated δ1⁸O and δD values in recent recharge sources, such as Nile water and irrigation return flow (δ1⁸O =  + 2.39 ‰, δD =  + 22 ‰ and δ1⁸O** = ** + 4.9 ‰, δD =  + 12.7 ‰, respectively), indicate enhanced surface water-groundwater interactions. The infiltration of surface water is accompanied by increased pollutant transport, highlighting the risk of contaminants percolating into deeper groundwater layers.

Figure 8 illustrates the spatial distribution of groundwater vulnerability using 1⁸O as a tracer for lateral percolation in the study area. The isotopic composition of groundwater samples provides insights into recharge dynamics and pollutant migration, highlighting zones susceptible to contamination due to surface water-groundwater interactions. Some areas are exhibiting elevated δ1⁸O values, this indicates active recharge areas, often associated with surface water infiltration from canals or irrigation return flow. These zones may experience higher pollution susceptibility due to the introduction of contaminants carried by surface runoff. Conversely, lower δ1⁸O values suggest older groundwater with reduced surface water influence, potentially indicating less vulnerability to recent contamination events.

**Fig. 9:**
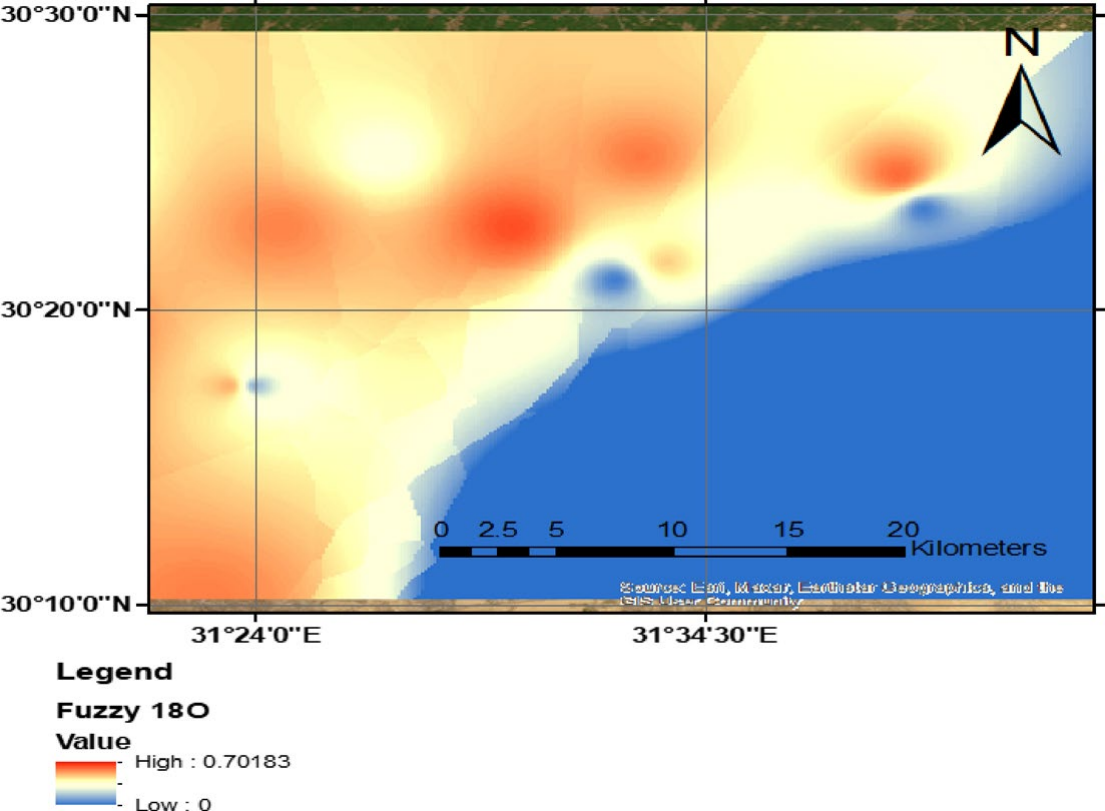
Spatial distribution of groundwater vulnerability using ^18^O as lateral percolation in the study area, (the Map was created using (the Map was created using QGIS (QGIS Development Team, 2025, Version: 3.34.6-Prizren. Available at: https://qgis.org).

The spatial pattern shown in Fig. 9 supports the identification of hydrological connectivity between surface water sources and groundwater reservoirs, enabling the characterization of pollutant transport mechanisms. Understanding these isotopic variations is essential for assessing contamination risks, improving groundwater management strategies, and implementing targeted mitigation measures.

The GOD index, which evaluates groundwater vulnerability based on aquifer characteristics, shows values ranging from 0.04 to 0.28 in the study area. These values indicate that the region predominantly falls within the negligible to medium susceptibility to pollution classification, emphasizing high protectability and effective pollutant retardability. Areas with the lowest GOD index values (≤ 0.10) are characterized by high protection capacity, suggesting minimal exposure to contamination risks. Regions with moderate GOD index values (0.20–0.28) may still possess some susceptibility, particularly in zones where aquifer confinement is less pronounced.

**Fig. 10:**
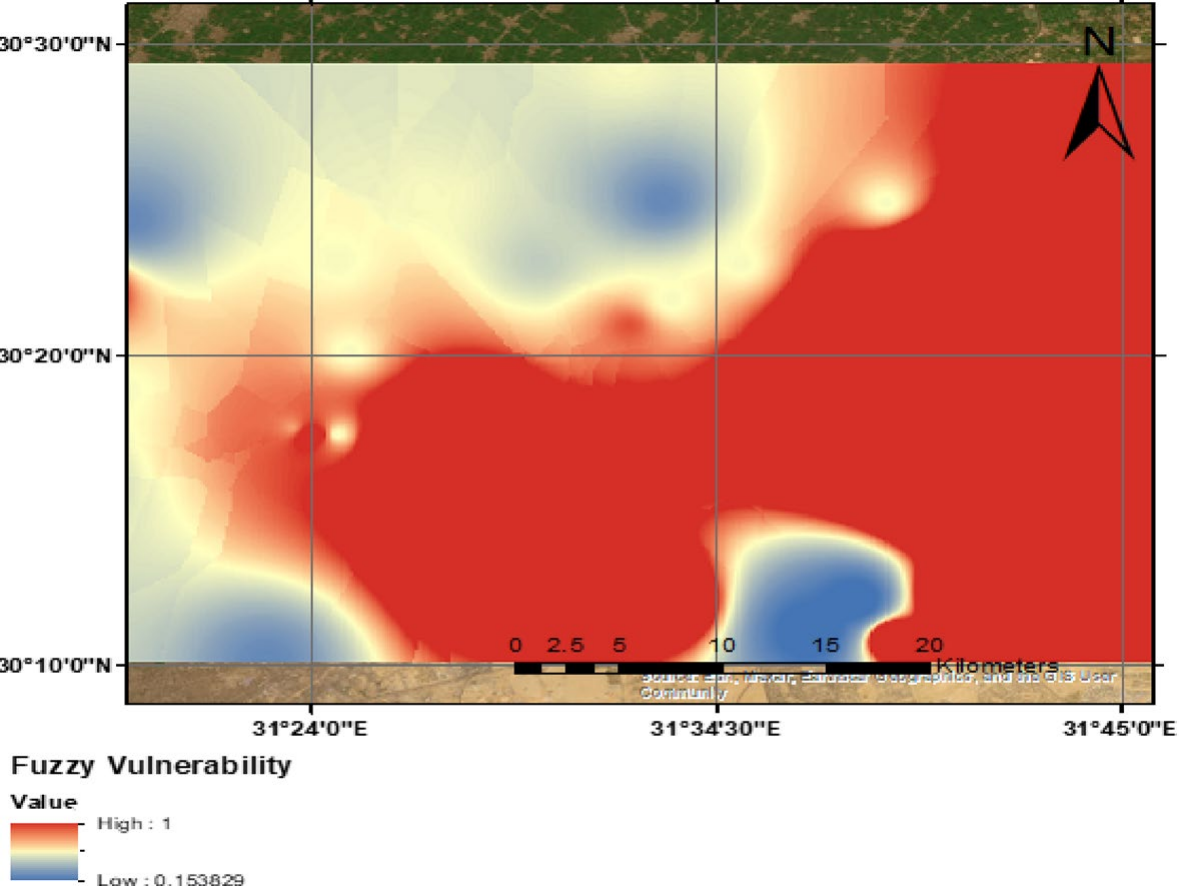
Spatial distribution of groundwater vulnerability using GOD in the study area, (the Map was created (the Map was created using QGIS (QGIS Development Team, 2025, Version: 3.34.6-Prizren. Available at: https://qgis.org).

The spatial distribution depicted in Fig. 10 confirms that aquifer characteristics play a dominant role in groundwater resilience, mitigating pollution threats in the study area. The presence of thick confining beds above the water table acts as a protective layer, preventing direct contamination from surface sources. In addition to the characteristics of the unsaturated zone that play a critical role in delaying pollutant infiltration, reducing the risk of rapid groundwater contamination. Clay minerals facilitate adsorption processes, capturing and attenuating pollutants before they reach the saturated zone of the aquifer. This attenuation mechanism ensures that contaminants undergo chemical and physical transformations, further limiting their mobility.

With respect to the protective attenuation mechanisms: the GOD index results indicate low susceptibility to pollution, largely due to clay-rich layers and thick confining beds above the water table, which slow contaminant infiltration and enhance adsorption. However, stable isotope data (oxygen-18 and deuterium) highlight zones influenced by recent recharge from surface water sources, such as Nile water and irrigation return flow, suggesting potential pathways for lateral pollutant migration beyond vertical attenuation. On the other hand, with Recharge Dynamics and Pollution Transport. Stable isotope composition reveals a wide range of δ^1^⁸O values reflecting different recharge sources and varying pollutant retardability. The presence of enriched isotope values in areas of recent recharge suggests higher vulnerability due to increased pollutant infiltration, particularly from surface canals. While the GOD index assesses vertical retardation capacity, stable isotope data confirm lateral migration, demonstrating that contamination risks are not solely limited to vertical transport but also influenced by surface–groundwater interactions. Spatially, the GOD index spatial distribution indicates greater protectability in regions with high clay content and low permeability, minimizing direct surface contamination. However, the eastern portion of the study area, influenced by high concentrations of pollution sources, demonstrates higher probabilities of pollutant migration, reinforcing the need to account for lateral flow mechanisms in vulnerability assessments.

#### Integrated approach for groundwater protection

The correlation between the GOD index vulnerability assessment and stable isotope interpretation provides a comprehensive understanding of groundwater susceptibility. While the GOD index provides valuable insight into aquifer confinement and attenuation potential, stable isotope analysis enhances the understanding of recharge dynamics and lateral connectivity. The integration of both methods enables:

- Better identification of pollution hotspots, considering both vertical infiltration resistance and lateral pollutant movement.
- More refined groundwater management strategies, ensuring adequate monitoring of recharge sources to prevent contamination spread.
- Comprehensive contamination risk assessments, incorporating hydrogeochemical properties and hydrodynamic behaviour to improve groundwater protection measures.

**Fig. 11:**
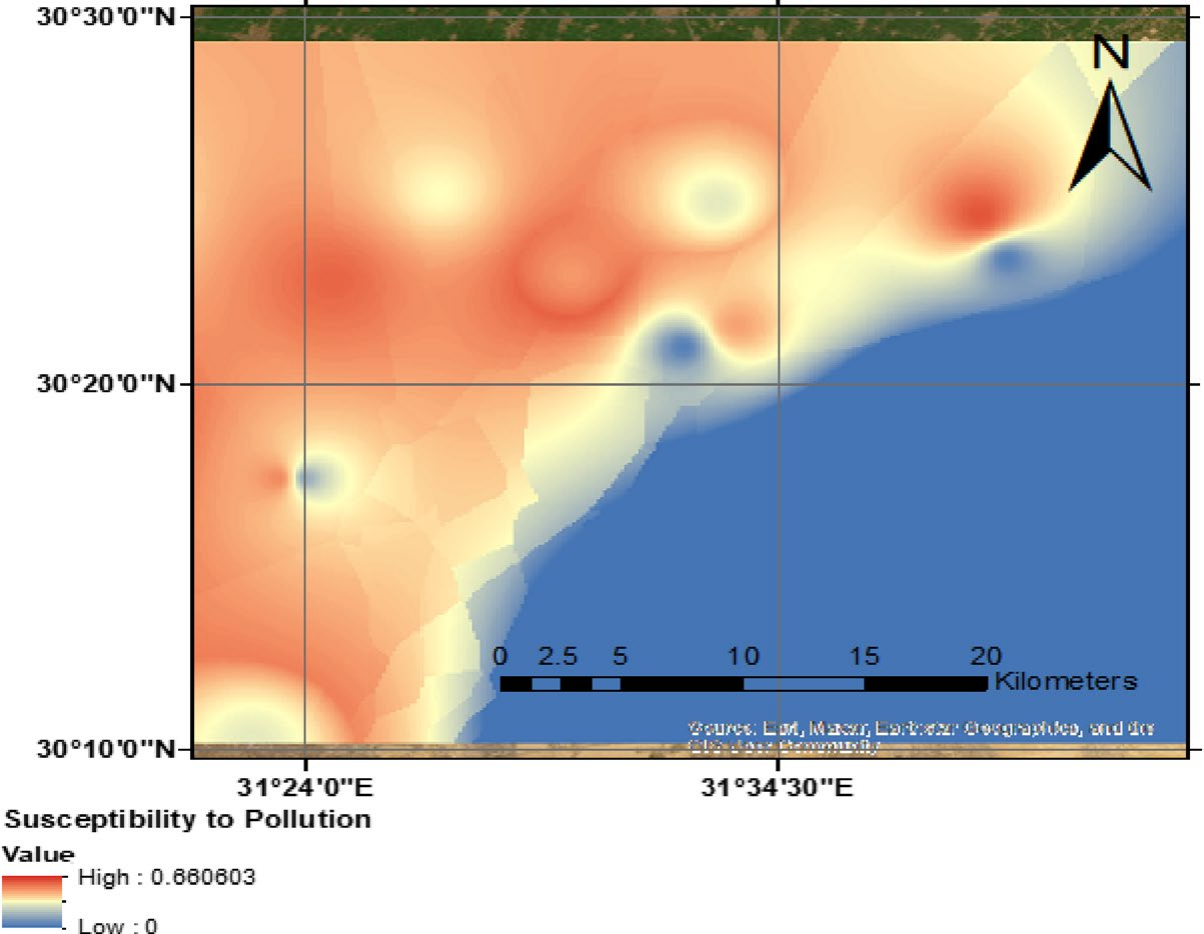
Spatial distribution of groundwater susceptibility in the study area, (the Map was created using Q-GIS).

The combined groundwater susceptibility map was estimated using and approach in the Fuzzy logic in GIS to take the two approached of contaminates percolation as a new lateral/vertical dimension in the study area as illustrated in Fig. 11.

### Groundwater contamination risk

Groundwater contamination risk is defined as the potential for anthropogenic activities to introduce pollutants into groundwater systems at levels exceeding acceptable thresholds. This risk is governed by the interaction between pollution loading, which quantifies the extent of contamination from human activities, and groundwater vulnerability, which reflects the intrinsic characteristics of the aquifer. The latter is dynamic, as it responds to variations in pollution loading.

In this study, the assessment of groundwater contamination susceptibility was conducted by integrating pollution loading data with vulnerability indices. The final contamination risk map (Fig. 11) was developed using the fuzzy overlay technique, combining groundwater vulnerability and pollution loading maps within a GIS platform. This approach allowed for the spatial identification of high-risk zones based on pollutant source distribution.

Fuzzy logic provides a computational framework for handling uncertainty in environmental assessments by assigning degrees of truth rather than binary classifications. In groundwater contamination risk analysis, the fuzzy overlay technique integrates pollution loading and groundwater vulnerability indices within a GIS platform, enabling a continuous risk evaluation. This method enhances spatial representation by allowing gradual transitions between risk categories, improving accuracy in identifying high-risk zones where pollutant sources overlap with sensitive aquifers. The generated fuzzy-based maps support contamination mitigation by visualizing pollution potential and prioritizing intervention efforts. By incorporating fuzzy logic into risk assessments, groundwater management strategies become more adaptive, facilitating effective decision-making for pollution control and resource protection.

**Fig. 12:**
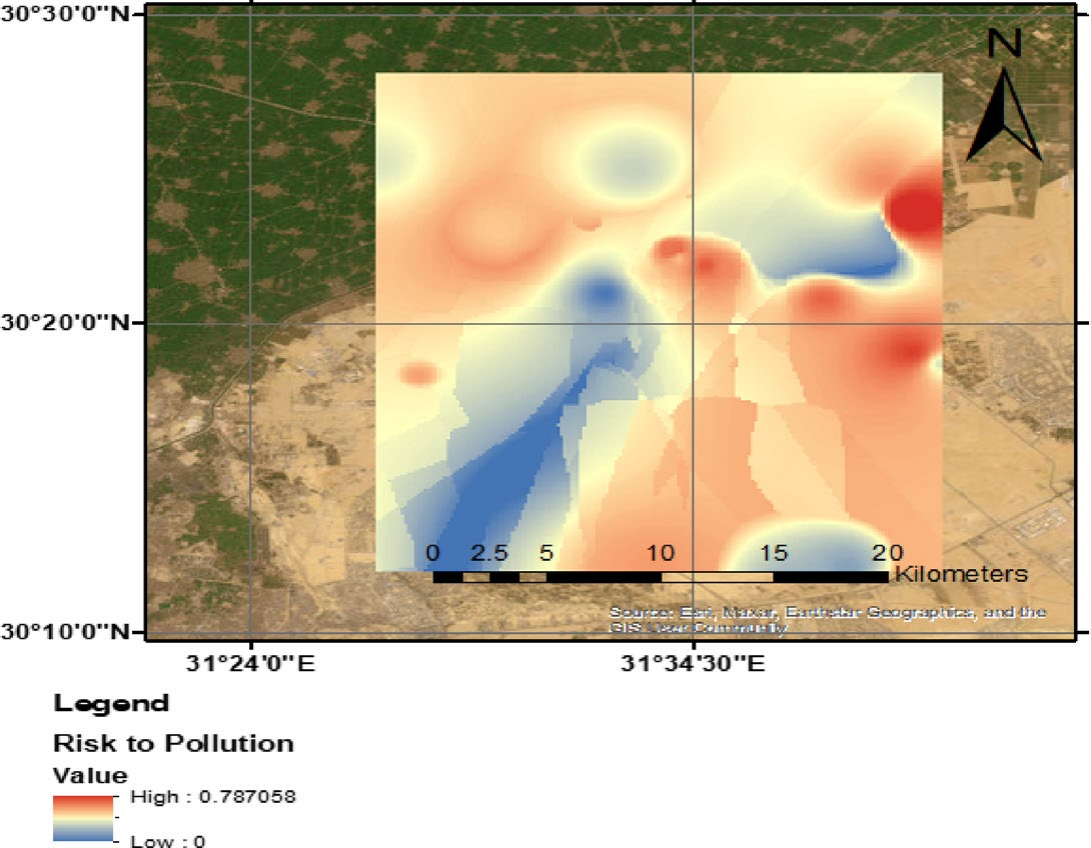
Spatial distribution of possible groundwater contamination risk in the study area, (the Map was created using QGIS (QGIS Development Team, 2025, Version: 3.34.6-Prizren. Available at: https://qgis.org).

The eastern portion of the study area exhibited a particularly high groundwater contamination risk due to the presence of sensitive aquifer conditions and significant pollution sources. As shown in Fig. 12, areas with elevated pollution loading and high intrinsic vulnerability were classified as very high and high-risk zones. These regions require careful monitoring and targeted mitigation strategies to prevent long-term groundwater degradation.

### Literature-based comparative validation

**Fig. 13:**
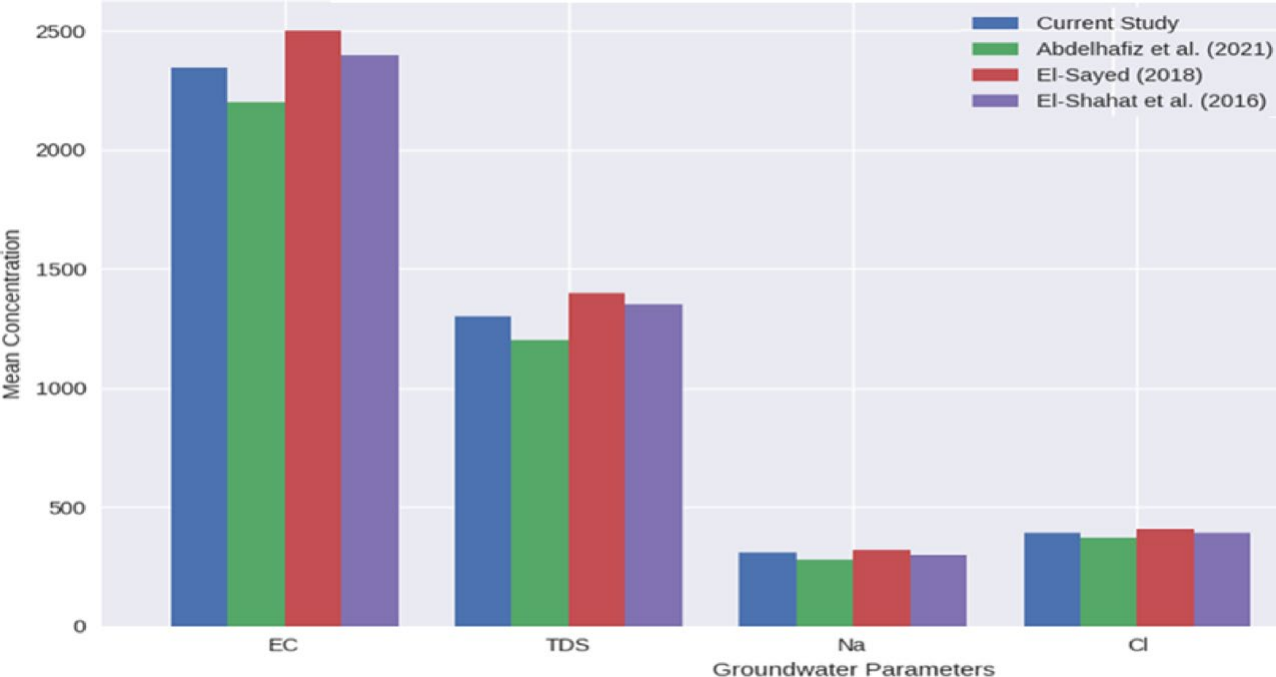
comparative literature-based validation with previous studies.

The results of this study are in line with earlier investigations in the same region, (Fig. 13) providing literature-based validation of the observed groundwater patterns. Recent studies in the study area,^33,52,53^, in which the high concentrations of salinity, nitrate, and trace metals in shallow aquifers were traced, mainly due to urban and agricultural sources. Their findings showed that spatial differences were more significant than seasonal changes. These consistent patterns confirm that spatial variability is the main factor affecting groundwater quality, and support the reliability of the current study’s results.

### Socio-economic implications for water use governance and land use planning

Groundwater management in this vulnerable and industrially stressed region requires integrated tools that combine hydrogeological, geospatial, and isotopic data. Vulnerability maps based on the GOD index can guide land-use planning by restricting high-pollution activities in sensitive zones, while pollution loading indicators help prioritize monitoring and intervention. Isotopic tracers (δ^1^⁸O, δ^2^H) provide insight into recharge sources and pollutant migration, supporting licensing decisions and verification of contamination pathways. Fuzzy logic models enhance classification of aquifer susceptibility by integrating multiple indicators. To support implementation, model outputs should be translated into spatial dashboards and updated regularly to inform transparent and adaptive groundwater protection.

The proposed framework has direct socio-economic relevance, particularly in areas undergoing rapid urbanization, agricultural expansion, and industrial growth. Vulnerability zoning supports strategic resource allocation, where low-risk areas may be prioritized for abstraction and high-risk zones require stricter controls. This enables balanced water use that considers both economic needs and long-term sustainability.

Transparent mapping and isotopic evidence improve public trust and support fair enforcement, encouraging community participation in groundwater protection. For industrial and agricultural sectors, operating in vulnerable zones may involve higher compliance costs, but these are offset by reduced long-term risks to water quality and infrastructure. Incorporating spatial outputs into national water management plans and land-use regulations ensures that groundwater protection remains technically sound, socially equitable, and economically viable.

### Implications for regulation and territorial planning

The integration of vulnerability mapping and isotopic analysis in this study provides a practical framework for groundwater governance in environmentally stressed and rapidly urbanizing regions. By combining GOD index scores, pollution loading indicators, and stable isotope tracers, the approach enables spatial identification of high-risk zones and contamination pathways, supporting both regulatory enforcement and territorial planning.

#### Zoning and land-use regulation

Vulnerability maps can be used to delineate protection zones where land development, industrial siting, and drilling activities are restricted based on aquifer susceptibility. Medium to high vulnerability areas should be prioritized for buffer zone designation, especially near recharge zones and surface water bodies with isotopically confirmed connectivity. Urban expansion and agricultural activities must be guided by these overlays to prevent pollutant loading in sensitive areas.

#### Monitoring and licensing

Isotopic signatures (e.g., elevated δ^1^⁸O and δ^2^H values) help verify recharge sources and detect anthropogenic influence, supporting pollution source attribution and regulatory compliance. Licensing for groundwater abstraction and wastewater discharge should incorporate both vulnerability scores and isotopic evidence to ensure sustainable aquifer use. Regular updates to the contamination risk map, informed by ongoing isotopic and hydrochemical monitoring, will support adaptive management and early warning systems.

#### Infrastructure and investment planning

Infrastructure investments, such as wastewater treatment upgrades and stormwater diversion, should be prioritized in zones with high pollution loading potential and confirmed lateral migration risks. Recharge enhancement projects must consider both vertical protectability (GOD index) and lateral vulnerability (isotope-based transport pathways) to avoid unintended contamination and ensure long-term aquifer resilience.

#### Policy integration and stakeholder engagement

The spatial outputs of this study can be translated into bilingual stakeholder maps and planning dashboards to support coordination among water authorities, urban planners, and environmental regulators. Integration with national water quality monitoring frameworks and land-use planning systems will promote policy coherence and operational uptake, ensuring that groundwater protection measures are technically sound, socially equitable, and economically viable.

### Limitations and uncertainty considerations

While the proposed framework offers a robust and spatially explicit approach to groundwater contamination risk assessment, several methodological limitations and sources of uncertainty should be acknowledged as following. The spatial resolution of sampling was constrained by logistical access and site permissions, particularly in industrial impacted zones. This may influence the granularity of vulnerability classification and pollutant loading estimates. Temporal coverage was limited to a single-season campaign, justified by the aquifer’s long residence time and documented geochemical stability in the study area. Isotopic data (δ^1^⁸O) were obtained from discrete sampling points without continuous monitoring, which may obscure transient mixing processes or seasonal recharge pulses. Remote sensing and land use classification relied on medium-resolution satellite imagery, potentially limiting the detection of small-scale or informal pollution sources. The framework was developed for the hydrogeologic and anthropogenic context of northeastern Greater Cairo. Application to other regions may require recalibration of vulnerability thresholds, isotopic baselines, and fuzzy logic rules. The integration of geospatial pollutant indicators is context-dependent and may not fully capture diffuse or undocumented sources in less monitored areas. To address these limitations, future research should expand sampling across multiple depths to capture temporal dynamics and vertical stratification. Incorporating additional tracers (e.g., δ^2^H, Tritium, nitrate isotopes) and continuous monitoring would refine recharge and transport models. It is recommended using machine learning classifiers and field-based contamination metrics.

## Conclusion

This work introduces a novel approach for assessing groundwater vulnerability to contamination and evaluating pollution risk with greater precision. In which land use and land cover analysis with pollution loading assessments, GOD index vulnerability evaluation, and stable isotope tracers were integrated comprehensively to assess groundwater contamination risks. Land use dynamics, including urbanization and agricultural expansion, significantly influence pollutant transport and groundwater susceptibility. The pollution loading index quantifies contamination potential, highlighting areas where anthropogenic activities exert pressure on groundwater quality.

The GOD index reveals that regions with high clay content exhibit low permeability, enhancing pollutant attenuation and reducing vulnerability. However, stable isotope analysis demonstrates that lateral percolation and recharge from surface water sources contribute to pollutant migration, affecting groundwater quality beyond vertical infiltration mechanisms. Elevated δ1⁸O and δD values in areas influenced by irrigation return flow and surface water interactions indicate increased contamination risks. The spatial correlation between land use, pollution sources, GOD index values, and isotopic signatures identifies high-risk zones, particularly in industrialized areas where pollutant accumulation and migration are intensified. Integrating these assessments enables a refined groundwater management strategy, prioritizing high-susceptibility regions for pollution mitigation and sustainable resource conservation. Continuous monitoring and adaptive land-use planning are essential for long-term groundwater protection.

Priority management strategies should focus on high-risk areas, emphasizing the regulation of future land development, industrial wastewater disposal, and drilling activities to mitigate contamination. Preventing the establishment of high-loading enterprises in highly vulnerable zones is essential to preserving groundwater quality. Furthermore, periodic updates of the groundwater contamination risk map are necessary for maintaining accuracy in risk assessment. The findings of this study serve as a foundation for decision-makers in groundwater management and land-use planning, facilitating the identification of high-risk zones and rationalizing pollution source distribution. The protective geological formations in the study area significantly reduce susceptibility, but continuous monitoring remains necessary to detect potential changes in aquifer conditions. Implementing land-use regulations, minimizing pollutant discharge near recharge areas, and promoting efficient wastewater management will further support groundwater conservation.

## Data Availability

All data generated or analysed during this study are included in this manuscript.
